# Placental Mesenchymal Stem Cell-Derived Extracellular Vesicles (PMSC-EVs) as an Innovative Therapy for Diabetic Wound Healing

**DOI:** 10.3390/ijms27094053

**Published:** 2026-04-30

**Authors:** Hady S. Omar, Amal Abdul-Hafez, Ranga Prasanth Thiruvenkataramani, Suraj Karanje, Sherif Abdelfattah Ibrahim, Sarah Jameel Mohammadi, Burra V. Madhukar, Said A. Omar

**Affiliations:** 1 Division of Neonatology, Department of Pediatrics and Human Development, College of Human Medicine, Michigan State University, East Lansing, MI 48824, USA; omarhady@msu.edu (H.S.O.); abdulhaf@msu.edu (A.A.-H.); thiruve5@msu.edu (R.P.T.); karanjes@msu.edu (S.K.); ibrahi22@msu.edu (S.A.I.); jameelmo@msu.edu (S.J.M.); madhukar@msu.edu (B.V.M.); 2 Regional Neonatal Intensive Care Unit, University of Michigan Health-Sparrow, Lansing, MI 48912, USA; 3 Institute of Biomedicine, Faculty of Health Sciences, University of Eastern Finland, FI-70210 Kuopio, Finland

**Keywords:** diabetes mellitus, wound healing, mesenchymal stem cells, extracellular vesicles, exosomes

## Abstract

Individuals with diabetes mellitus (DM) experience impaired wound healing, where the healing process is often compromised by a complex, hostile microenvironment characterized by persistent inflammation, high oxidative stress, and dysfunctional angiogenesis. The hyperglycemic environment damages the blood vessels and disturbs the normal hypoxia-induced upregulation of vascular endothelial growth factors, causes poor vascularization and insufficient production of new blood vessels, and leads to impaired perfusion and thickened and dysfunctional capillary basement membranes, which reduce blood flow to the wound, leading to delayed wound healing. Mesenchymal stem cell (MSC)-derived extracellular vesicles (EVs) are the main effectors of intercellular communication and have emerged as a potent cell-free strategy for the acceleration of tissue repair. MSC-EVs can be isolated from various adult tissues, but increasing evidence suggests that placental MSC-derived EVs (PMSC-EVs) possess distinct clinical and biological advantages for enhancing diabetic wound healing. Placentas are unique cell sources for PMSCs, which can be easily acquired non-invasively from a discarded placenta and is ethically acceptable, and have superior proliferative capacity. The cargo of PMSC-derived EVs contains macromolecules such as proteins, mRNA, miRNA, and lipids, which may be tailored for fetomaternal tolerance and translates to unmatched immunomodulatory potential for resolving chronic diabetic inflammation. The PMSC-derived EVs also aid in enhancing multiple pathways, including modulation of inflammation, angiogenesis, and epithelial proliferation, that lead to increased wound healing. This article will highlight the unique advantages, specific mechanisms, and limitations of PMSC-derived EVs as an innovative non-cellular therapeutic modality in restoring vital repair processes and enhancing diabetic wound healing.

## 1. Introduction

Diabetes mellitus (DM) is a chronic metabolic condition characterized by persistent high blood glucose levels and is associated with serious long-term complications. One of the complications that individuals with DM experience is impaired wound healing. Diabetic wounds heal at a prolonged rate compared with non-diabetic patients. Adults and children with poorly controlled diabetes are at increased risk of poor wound healing due to diabetic neuropathy, poor circulation, modified inflammatory response, and persistently elevated blood glucose, which impair neutrophil function and delay the healing process. The imbalance of blood supply in diabetic patients is associated with ischemic conditions [1,2,3]. In these diabetic patients, angiogenesis is often compromised due to inflammation and oxidative stress; therefore, the body tissues do not receive an adequate blood supply, leading to insufficient nutrients and a prolonged wound healing time.

## 2. Methods

This systematic review was developed following established methodological standards. This review contains studies involving in vitro hyperglycemic wound models, animal models of diabetes with cutaneous wounds, and human subjects with diabetic wounds or diabetic foot ulcers. The intervention in these studies was the use of extracellular vesicles (EVs), including exosomes or small EVs, derived from placental mesenchymal stem/stromal cells (MSCs), including MSCs isolated from the chorionic plate, decidua basalis, amniotic membrane, Wharton’s jelly, and whole placenta. This was compared with untreated or vehicle-treated controls. These studies used direct MSC therapy, with EVs derived from other MSC sources. The primary outcomes were wound closure rate (%), time to complete wound healing, and degree of re-epithelialization. The secondary outcomes were angiogenesis markers (e.g., VEGF), collagen deposition and organization, inflammatory cytokines (e.g., TNF-α, IL-6, and IL-10), macrophage polarization markers (M1/M2), oxidative stress markers, and adverse events or safety outcomes. These studies included original research articles; in vitro, in vivo, or clinical studies; explicit use of placental MSC-derived EVs; quantitative wound healing outcomes; and adequate EV characterization consistent with current extracellular vesicle standards. The following studies were excluded: editorials, commentaries, conference abstracts without full data, studies not involving diabetic wound models, and studies lacking EV characterization data. A systematic literature search was performed in PubMed, Scopus, and Web of Science from January 2010 to January 2026 using predefined search terms combining: placental mesenchymal stem cells; extracellular vesicles; and diabetic wound healing. Inclusion criteria comprised original in vitro, in vivo, and clinical studies evaluating the therapeutic effects of placental MSC-derived EVs on diabetic wound healing. The reference lists of included studies were manually screened to identify additional eligible studies. The retrieved records were exported into Mendeley reference management software, and duplicates were removed. Two independent authors screened titles and abstracts for eligibility. Full-text articles were subsequently assessed against the predefined inclusion and exclusion criteria. The disagreements between the two authors were resolved through discussion or consultation with a third author. The studies were required to demonstrate characterization of EVs in accordance with the Minimal Information for Studies of Extracellular Vesicles (MISEV) guidelines published by the International Society for Extracellular Vesicles [4,5]. The literature search identified 1229 records. After screening and removal of duplicates, a total of 124 studies met the inclusion criteria and were included in this review.

## 3. Pathophysiology of Diabetic Wound Healing

The wound healing process goes through four stages that involve several biological processes [3]. The first stage is hemostasis followed by the induction of a limited inflammatory process that continues for up to 4 days. The inflammatory process decreases the proliferation of several cell types required to rebuild the damaged tissues, including the endothelial cells for angiogenesis, the fibroblasts for connective tissue formation, and the keratinocytes for re-epithelization. The final step is remodeling, where the tissue resumes its original architecture. The delayed wound healing in diabetic patients occurs due to disruption of several of these processes.

The initial limited inflammatory response to the wound is very central in the wound healing process. However, an extended or exaggerated inflammatory response delays wound healing through several mechanisms that mainly involve the production of oxidative stress and release of paracrine factors that hinder migration and proliferation of the cellular components of wound healing such as endothelial cells, fibroblasts, and keratinocytes. The exaggeration of the inflammatory response in DM occurs due to hyperglycemia, which induces oxidative stress, leading to the enhancement of pro-inflammatory factors such as TNF-α, IL-1β, IL-6, and IL-8 and suppression of anti-inflammatory factors such as vascular endothelial growth factor (VEGF) and Transforming Growth Factor beta-1 (TGF-β). Enhancement of these pro-inflammatory factors results in excess recruitment of neutrophils and macrophages to the site, contributes to prolonged inflammation, and suppresses the anti-inflammatory cytokines, leading to an impaired transition from inflammation to proliferation, which, in turn, delays wound healing [6]. Another important factor that limits the inflammatory response is the transition of the pro-inflammatory macrophages (M1) to the anti-inflammatory macrophage (M2). Some anti-inflammatory cytokines such as IL-4 play an important role in promoting this transition [7]. M2 polarization is associated with tissue repair, regeneration, and wound healing [8]. In diabetic wounds, chronic inflammation and impaired macrophage function hinder healing. IL-4 supports the shift of the immune response toward a reparative phenotype, counteracting this dysfunction. IL-4 enhances angiogenesis, fibroblast activation, and collagen deposition, all of which are critical for wound closure and diabetic wound healing [8]. VEGF is a key mediator of angiogenesis because it helps in the formation of new blood vessels [9]. In hypoxic conditions, VEGF is upregulated to promote angiogenic activity and increase blood flow. Low oxygen levels in cutaneous wounds activate the transcription factor hypoxia inducible factor-1 (HIF-1), which drives the transcription of the VEGF gene [9,10]. One of the issues with diabetic patients is that the hyperglycemic environment causes a high production of reactive oxygen species (ROS), leading to inflammation and oxidative stress, blood vessels damage, and disruption of the normal hypoxia-induced upregulation of VEGF [9,10]. The deregulation of VEGF causes delayed angiogenesis, poor vascularization, insufficient production of new blood vessels, impaired perfusion, and thickened and dysfunctional capillary basement membranes, which reduce blood flow to the wound, leading to delayed wound healing [11]. In addition, the high glucose levels impair nitric oxide production, which reduces vasodilation, further restricting blood flow. Persistent inflammation increases ROS, and active stress further delays healing [12].

Furthermore, hyperglycemia is associated with fibroblast dysfunction and cell death, which play a leading role in delayed healing by diminishing collagen activity and reducing extracellular matrix formation, thereby impairing the restoration of the skin barrier [13]. This fibroblast death occurs due to ferroptosis that is induced by neutrophil extracellular traps (NETs), the activity of which is increased due to hyperglycemia [13]. Another mechanism that was involved in the reduction of fibroblast proliferation is the reduction of platelet-derived growth factor (PDGF) that occurs in diabetes [14]. There is a significant reduction in PDGF A and A-type receptor expression in diabetic animals. In addition, systemic glucocorticoid treatment causes a severe defect in wound repair accompanied by reduced expression of PDGFs A and B and of the B-type receptor in the early phase of wound healing [14]. A certain expression level of PDGF and its receptors is essential for normal wound repair, and there is a possible beneficial effect of exogenous PDGF in the treatment of wound healing disorders [14].

Finally, the lack of keratinocyte proliferation, migration, and adhesion caused by hyperglycemia is preventing re-epithelialization and delaying barrier restoration. One of the factors that affect keratinocyte re-epithelization is the matrix metalloproteinases (MMPs). Imbalances of these MMPs either increase or decrease their activity, leading to delayed re-epithelization and delayed wound healing [15]. The forehead box O1 (FOXO1) transcription factor plays an essential role in the regulation of MMPs in normal and diabetic wound healing. It also affects keratinocyte proliferation and migration through other factors such as TGF-*β* [16].

From the above discussion on wound healing, it is clear that several mechanisms are involved in delayed wound healing induced by diabetes. The multiple mechanisms involved in wound healing and their disruption in diabetic patients suggest that multiple therapeutic modalities are necessary in the successful treatment of chronic wounds in diabetic patients. Currently available diabetic wound healing therapies, including wound debridement and dressing, transcutaneous electrical nerve stimulation (TENS), nanomedicine, shockwave therapy, hyperbaric (HBOT) and topical oxygen therapies (TOT), photobiomodulation (PBM), and biomaterial such as hydrogels, have been used in the management of chronic diabetic wounds, and they mainly focus on wound management but do not target the underlying disrupted physiological mechanisms that delay wound healing in diabetic patients [17,18].

## 4. Mesenchymal Stromal (Stem) Cells (MSCs)

Regenerative stem cell research and its potential role in regenerative medicine, including diabetic wound healing, is expanding. MSCs are multipotent stem cells that can differentiate into other types of cells with specific growth and differentiating factors. Stem cell-derived extracellular vesicles (EVs) represent one of the main effectors of intercellular communication and might play a key role in diabetic wound healing. In this paper, the potential use of placental MSC-derived EVs as a therapeutic option to improve diabetic wound healing will be reviewed. We will explore the potential mechanisms of action and efficacy of these EVs in improving wound healing during in vitro and in vivo studies in DM models.

### 4.1. MSCs for Tissue Regeneration

MSCs have been shown to regulate the immune system and have high potential for application in regenerative medicine. However, the lack of standardization and heterogeneity encompassing MSCs in cell isolation hinders their clinical therapeutic application. Several efforts have been applied towards this standardization, including the proposing of minimal criteria by the International Society for Cellular Therapy (ISCT) for the definition of human MSCs to increase consistency in their definition and verification. These criteria include the adherence to plastic in standard culture conditions; the expression of certain surface markers—CD105, CD73, and CD90—and absence of others:CD45, CD34, CD14, CD11b, CD79a, CD19, and HLA-DR; and the ability to differentiate into osteoblasts, adipocytes, and chondroblasts [19]. One of the main advantages of MSCs is their mechanism of action by paracrine cell signaling that induces a behavioral change in neighboring cells.

The beneficial effects of MSCs were initially thought to be due to their ability to home, engraft, and differentiate at the site of injured tissues. However, a small percentage of transplanted cells are estimated to reach the target tissues, with most of the cells trapped in the liver, spleen, and lungs. It is currently proposed that MSCs exert their therapeutic effects through the secretion of paracrine bioactive cargo, which can be either directly released to the surrounding environment or contained in EVs (MSC-EVs) [20]. Recent studies have proven that these MSC-EVs have regenerative capacity that is comparable to their cells of origin [21]. We have previously shown that placenta- and umbilical cord blood-derived small EVs play an important role in tissue regeneration such as the expansion of hematopoietic stem cells, prevention of LPS-induced lung epithelial cell injury, and, as possible therapy, for prevention of bronchopulmonary dysplasia (BPD) [22,23,24].

EVs derived from stem cells (e.g., adipose-derived mesenchymal stem cells) have shown promise in modulating immune responses and promoting tissue regeneration in diabetic wounds. These EVs can be engineered or naturally enriched with anti-inflammatory cytokines such as IL-4 or IL-4-induced miRNAs, enhancing their therapeutic efficacy. EVs carrying miRNAs that upregulate IL-4 signaling have been shown to promote M2 macrophage polarization and improve wound healing.

### 4.2. Major MSC Sources in Regenerative Medicine

MSCs from multiple sources have been previously studied in wound healing such as bone marrow MSCs (BM-MSCs), adipose-derived MSCs (AD-MSCs), umbilical cord/Wharton’s jelly MSCs (UC/WJ-MSCs), and placental MSCs (PMSCs). These different sources have their own biological profiles, secretomes, and functional properties that influence wound healing efficacy. Bone marrow MSCs (BM-MSCs) are the most studied source, with extensive historical data for their use. BM-MSCs have a good paracrine immunomodulatory effect and are effective in enhancing diabetic wound healing by reducing proteolysis and correcting collagen I levels through an increase in the expression of activated matrix metalloproteinase (MMP-9) in an miR-29b-dependent manner [25]. Their limitations include lower proliferative capacity compared with perinatal sources because donor age affects potency, with older donors having reduced function and less angiogenic signaling than placental sources. AD-MSCs are abundant, easily accessible with effective immunomodulatory effects, and enhance fibroblast migration and collagen synthesis in wound healing models [26]. Their limitations include moderate angiogenic activity compared with perinatal MSCs, with variable potency depending on the donor metabolic state. UC/WJ-MSCs have high proliferative capacity, low cellular senescence, potent immunomodulation and angiogenic paracrine signaling, and in general outperform BM-MSCs in inflammatory and proliferative capacity for healing [27]. Their limitations include heterogeneity between UC vs. WJ subtypes, and they require perinatal gestational tissue screening and processing. Several previous reviews have outlined the advantage of using MSCs and EVs from adult sources such as bone marrow and adipose tissue in diabetic wound healing [28,29,30,31]. In this review, we emphasize the advantages of PMSCs and their derived EVs in diabetic wound healing. There is no adequate number of studies to directly compare the effect of adult MSC-derived EVs with PMSC-derived EVs. We summarize the available studies in this review. PMSCs include MSCs from different placental tissue sections, human amniotic epithelial cells (hAECs), and human umbilical cord mesenchymal stem cells (hUC-MSCs), among others.

## 5. Placental MSCs (PMSCs)

MSCs that are derived from the gestational tissues (umbilical cord and placenta), in contrast with sources such as adipose tissue, bone marrow, and peripheral blood, have the advantage of being a feasible unlimited source of stem cells. The use of these tissues has minimal ethical dilemmas and causes no harm to the donor as they are considered medical waste. The placenta-derived MSCs show high plasticity, rapid proliferation, low immunogenicity, low risk of infection, and high stemness potential related to the early embryological origin of the placenta [32,33,34]. These PMSCs express stem cell transcription factors (Oct4, SOX2, and Nanog) without the risk of teratogenicity reported with embryonic stem cells [35]. In addition, the placental stem cells have unique immunomodulatory and immunosuppressive characteristics [36,37]. In addition, PMSCs can be isolated non-invasively compared with other sources such as bone marrow or adipose tissues that utilize invasive and highly uncomfortable procedures to acquire the tissues from the patients, which hinder their long-term use [38]. The use of the patient’s own MSCs, in which the number and activity declines with age, can have impact on the viability of the cells because of specific disease or genetic conditions [39].

**Figure 1:**
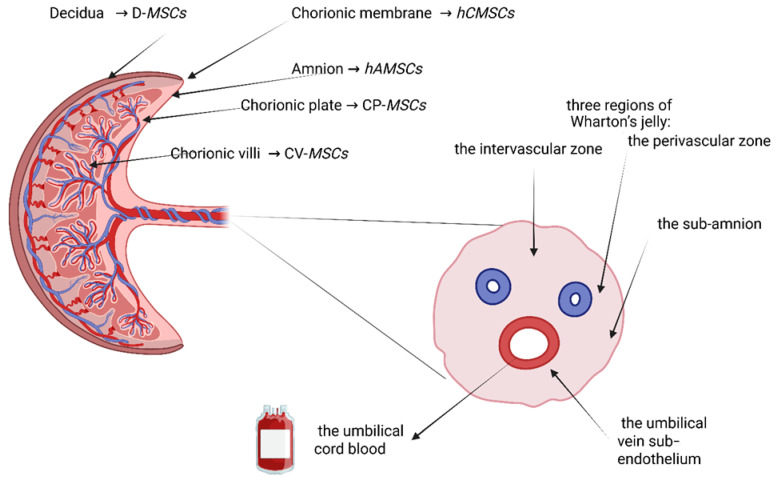
Source of MSCs from gestational tissues. The above figure shows the umbilical cord compartments. MSCs can be obtained from these regions: (1) from freshly prepared mononuclear cells from umbilical cord blood; (2) from the umbilical vein subendothelial layer; and from Wharton’s jelly: (3) after enzymatic digestion of the outer layers of umbilical vessels such as the perivascular region, (4) the intravascular space, and (5) the sub-amnion region.

MSCs have been isolated from several placental regions, as illustrated in Figure 1, including the fetal membranes, which yield human amniotic mesenchymal stromal cells (haMSCs) and human chorionic mesenchymal stromal cells (hCMSCs) [40]. In addition, they have been found in different regions of the decidua (D-MSCs) [41] and the fetal chorionic villi (CV-MSCs) [42]. In the umbilical cord, MSCs have been obtained from five compartments: the umbilical cord blood, the umbilical vein sub-endothelium, and three regions of Wharton’s jelly: the perivascular zone, the intervascular zone, and the sub-amnion [40].

### 5.1. Biological and Functional Activities of PMSCs

Gestational tissues (the placenta and umbilical cord) are highly vascular and developmentally early organs. They serve as a reservoir of stem cells, providing various types of placental mesenchymal stem cells (PMSCs) such as human umbilical cord mesenchymal stem cells (hucMSCs), human amniotic mesenchymal stem cells (hAMSCs), and human amniotic epithelial cells (hAECs). PMSCs express primitive molecular signatures, are expandable, demonstrate robust self-renewal and pluripotency, show lower expression of immune activation markers, and maintain minimal MHC class II upregulation even after stimulation, consistent with enhanced hypoimmunogenicity compared with adult MSCs [43]. The high self-renewal and low immunogenicity of PMSCs make them an attractive clinical therapeutic source for allogeneic use in chronic wounds. In contrast to adult sources of BM-MSCs and AD-MSCs, PMSCs have a greater proliferative capacity, immunomodulation, and secrete more types of growth factors [44,45]. PMSCs modulate secretion profiles of anti-inflammatory factors in animal studies of rat models, and their implantation reduced inflammatory responses and accelerated wound closure [46]. In diabetic wounds, dysregulated inflammation and chronic NF-κB activation impair healing. PMSCs co-cultured with dermal fibroblasts inhibited LPS-stimulated NF-κB activation, demonstrating intrinsic anti-inflammatory effects [46]. This inhibition of chronic inflammation is crucial in diabetic wounds, which are characterized by persistent pro-inflammatory signaling.

### 5.2. Angiogenic Signaling of PMSCs

One of the strongest reported advantages of PMSCs is strong angiogenesis support, which is a critical process impaired in diabetic wounds. PMSCs enhance microvessel density in wound beds, and transplanted PMSCs localize to wound tissue and incorporate into the vasculature, suggesting both paracrine and potential direct structural contributions to wound healing. PMSCs compared with adult MSCs (e.g., BM-MSCs or AD-MSCs), which predominantly rely on paracrine support, secrete bioactive levels of VEGF, HGF, bFGF, TGF-β, and IGF-1, which synergistically promote angiogenesis. PMSCs may participate in vascular regeneration more directly and robustly, a key factor in oxygen delivery and nutrient supply to ischemic diabetic wounds. This support that PMSCs are a potential angiogenesis cell therapy for repair-resistant chronic wounds in diabetic patients [47].

## 6. Advantages of Using EVs over Whole-Cell Therapy

MSC-derived EVs are vesicles released by stem cells and include small EVs or exosomes (~30–150 nm). These small EVs, which are well studied compared with other forms of EVs, originate from endosomal multivesicular bodies and are released when they are fused with the plasma membrane. Microvesicles (~100–1000 nm) bud directly from the plasma membrane, and apoptotic EVs are released during programmed cell death and can have unique signaling roles but are less studied in wound healing. These EVs can originate from multiple sources including BM-MSCs, AD-MSCs, UC-MSCs, WJ-MSCs, neonatal tissues, oral mucosa, menstrual blood, and iPSC-derived MSCs. These EVs have distinct cargo profiles reflecting their tissue of origin [48,49]. EVs have the potential to be used in regenerative medicine due to their unique properties in comparison with whole-cell therapy. EVs offer highly reduced immune rejection, tumorigenicity, and are easier to manage, making them a viable option to explore a more efficient way for wound healing. EVs are acellular, released into extracellular space from cells [50]. This distinct aspect of EVs makes them have a low risk of immunogenicity or immune rejection due to EVs evading the host immune system, leading to low chances of complications and efficiency in its healing properties [50]. Whole-cell therapy carries the risk of tumor formation because of cell proliferation. EVs do not have this problem because they lack the self-replicating capabilities [51].

## 7. Placental MSC (PMSC)-Derived EVs in Diabetic Wound Healing

**Figure 2:**
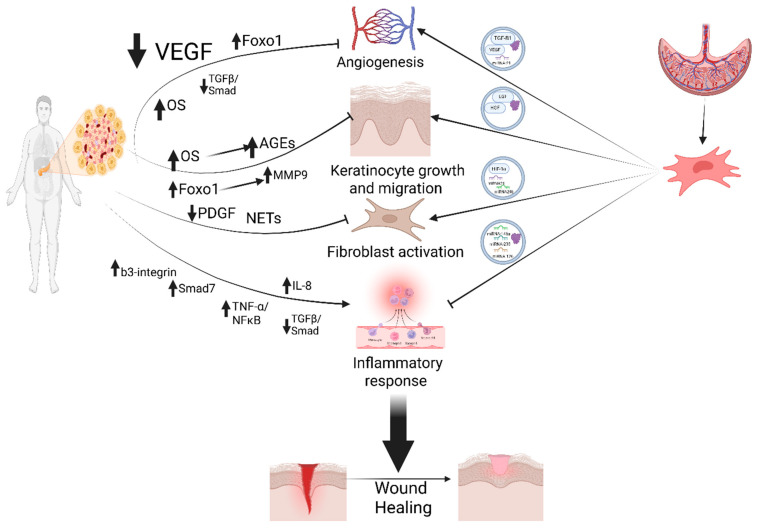
PMSC-derived EVs accelerate diabetic wound healing by delivering regulatory miRNAs and restoring cytokine balance, suppressing chronic inflammation, promoting macrophage polarization, and enhancing angiogenesis. Created in BioRender. Ibrahim, S. (2026) https://BioRender.com/9q5ceoj.

PMSC-EVs are an attractive alternative source isolated from the placenta, which is an organ that is essential for fetal development and “fetomaternal” tolerance. PMSCs are abundant, ethically uncomplicated to acquire, and possess unique biological properties, and their unique perinatal profile may make them superior cell free therapeutic modality. The cargo of placental MSC-derived EVs includes a variety of components such as proteins, mRNA, miRNA, and lipids, which may play a beneficial role in enhancing diabetic wound healing as illustrated in Figure 2.

### 7.1. Advantages of Placental MSC-EVs vs. Adult MSC-EVs

Many studies evaluate either placental PMSC-EV or adult MSC-EV sources such as BM or AD independently, but head-to-head comparative studies are relatively rare. Specific studies highlighted why placental or fetal sources may provide superior cargo in certain therapeutic areas such as immunomodulation, tolerance, and indicated that PMSCs are uniquely influenced by the placenta’s role in “fetomaternal” tolerance, and unlike adult BM-MSCs, PMSCs express Human Leukocyte Antigen-G (HLA-G), a non-classical MHC class I molecule that inhibits Natural Killer (NK) cell function [44,52,53]. This unique immunological profile is reflected in their EVs, potentially offering stronger immunomodulatory effects, relevant for autoimmune or inflammatory conditions. The existing comparative research studies are primarily in vitro and suggest that adipose-derived MSC-EVs generally exhibit superior angiogenic potency and protein expression compared with BM-derived EVs [54]. One of these rare studies showed that canine PMSC-derived EVs exhibited robust expansion capacity and high purity in culture compared with ADMSC-derived EVs [55]. Their primitive origin allows them to achieve greater cell numbers in fewer passages, reducing the risk of ex vivo senescence influencing the secreted EV pool, a common limitation when using adult ADMSC-EVs. PMSC-derived EVs had comparable immunomodulatory effects to ADMSC-derived EVs [55]. PMSC-derived EVs had significantly lower TNF-α, with similar IL-2 and IL-10 levels, and had a greater ability to inhibit proliferation than the ADMSC-EVs [55].

### 7.2. Role of miRNA Cargo in PMSC-Derived EVs in Diabetic Wound Healing

Recently, miRNA was recognized to play an essential role in the inflammation involved in wound healing and is being considered as a new therapeutic direction for wound treatment [56]. The targeted delivery of different miRNAs through MSC-derived EVs to treat diverse diseases is recently viewed as a safe and effective miRNA delivery system [57]. Different miRNA released in PMSC-EVs improve wound healing through tackling different mechanisms that delay wound healing in diabetes as seen in Figure 2. EV-mediated microenvironmental changes are mediated by miRNAs’ exchange of bio-information between neighboring cells. miRNAs in EVs are remarkably stable, likely because of their unique structure that protects them against harsh degradation. The role of the miRNA processing in immune cell development and inflammatory responses modulates the expression of inflammation-related genes, reduces delayed inflammation, and enhances wound healing.

Prominent microRNA (miRNA) such as miR-21 enhance fibroblast migration, angiogenesis, and reduces apoptosis, which are factors that are highly deregulated in diabetes. Dysregulation of miR-21 or miR-181 induces a chronic inflammatory state; these miRNA are key in controlling the balance between initial pro-inflammatory and later immuno-regulatory, anti-inflammatory responses [58,59]. MSC-derived small EVs (exosomes) mitigate oxidative stress-induced senescence in endothelial cells via regulation of miR-146a. MiR-146a promotes diabetic wound healing via macrophage M1/M2 polarization and is correlated with anti-inflammatory properties, which helps reduce inflammation by suppressing pro-inflammatory cytokines such as IL-6, IL-1β, and TNF-α while simultaneously increasing anti-inflammatory cytokines such as IL-10 [60,61,62]. Other miRNAs such as MiR-17-5p improve diabetic wound healing by enhancing angiogenesis through the induction of VEGF in endothelial cells [63].

MicroRNA (miR-223), as a regulator of innate immunity, suppresses the NF-kb signaling pathway, which further reduces inflammation [64,65,66]. MiR-223-3p promotes angiogenesis and helps the immunomodulatory effect to promote wound healing by targeting FOXO1 [67]. MiR-29b-3p is another example of miRNA that is a part of the MSC-derived EVs’ cargo and has been shown to promote collagen synthesis, which is a key element in skin wound healing [68,69]. These studies show that EVs from MSCs, including PMSCs, have the capacity to deliver miRNAs known to promote wound healing.

### 7.3. Role of Growth Factors in PMSC-Derived EVs in Diabetic Wound Healing

In addition to miRNA, PMSC-EVs deliver growth factors that would support wound healing in diabetic patients as illustrated in Figure 2. VEGF is one of the factors that mediate the stimulation of angiogenesis by umbilical cord EVs via protein transfer and is a growth factor that stimulates the proliferation of endothelial cells and is controlled by HIF-1α that is stabilized in a hypoxic environment [70,71]. Both VGEF and HIF-1α further promote angiogenesis, leading to the formation of blood vessels, which aids in bringing nutrients and oxygen into the wound site.

Another growth factor that is contained in MSC-derived EVs is TGF-β1/SMAD, which upregulates miR-132 and promotes M2 macrophage polarization, improving wound healing and reducing scar tissue formation [72,73,74,75,76]. Hepatocyte growth factor (HGF) is also reported to be included in the MSC-EVs. HGF stimulates epithelial cell proliferation and migration to stabilize endothelial barrier function and promotes epithelial repair and neovascularization during wound healing [77,78]. MSC-derived EVs have also been shown to contain epidermal growth factor (EGF) within their cargo. EGF is recognized to influence the keratinocyte components that regulate wound epithelialization speed [79,80]. This potential growth factor delivery via MSC-derived EVs can further enhance keratinocytes and fibroblast activation as well as promote collagen and fibronectin deposition to support wound closure and healing. UC-MSC-EV miR-17-5p could downregulate PTEN via activating the AKT/HIF-1α/VEGF signaling pathway to promote angiogenesis, thus contributing to the healing of diabetic wounds.

### 7.4. Role of PMSC-Derived EVs in Promoting Angiogenesis in Diabetic Wound Healing

PMSC-derived EVs are enriched in pro-angiogenic cargo including mRNA for angiogenic signals (VEGF-A and eNOS), miRNA for endothelial proliferation (miR-126, miR-21, miR-210, and miR-132), growth factors for vessels formation (VEGF, FGF2, and PDGF), protein for vascular stabilization (angiopoietin-1 and HGF), and lipids for vesicles signaling (Sphingomyelin and ceramide). Placental EVs are particularly enriched in hypoxia-responsive miRNAs, which are critical for angiogenesis, significantly increase the number and complexity of capillary-like structures, and enhance the expression of key pro-angiogenic factors, including VEGF, angiopoietin-1, and angiopoietin-2 [81]. The VEGF pathway is the central regulator of angiogenesis. PMSC-EVs increase VEGF signaling via delivery of VEGF mRNA, upregulation of VEGFR2 expression, and activation of PI3K/AKT signaling. This leads to endothelial cell proliferation, migration, and tube formation [82]. In diabetic tissues, hyperglycemia destabilizes HIF-1α, a critical trigger for angiogenesis, impairing the hypoxic response and reducing angiogenic capacity. PMSC-EVs restore HIF-1α signaling by delivering regulatory microRNAs and proteins that inhibit HIF degradation pathways and increase the expression of VEGF, PDGF, angiopoietin-1, and angiopoietin-2 [81]. In addition, hypoxia-induced-EVs produced by human umbilical cord mesenchymal stem cells (HUCMSCs) significantly promote angiogenesis through activation of the HIF-1α pathway, modulate macrophage polarization, and attenuate cellular oxidative stress, possibly through the delivery of specific miRNAs and proteins with a theoretical basis and potential application to develop novel therapeutic strategies against diabetes-related tissue injury [83]. This restoration of hypoxia signaling enhances activation of pro-angiogenic transcriptional signals, expression of angiogenic growth factors, and promotes vascular regeneration. In addition, UC-MSC-derived EVs had a dose response effect in dermal fibroblast function and supported their potential in wound healing applications. These EVs at lower concentrations (25–50 µg/mL) significantly enhanced fibroblast metabolic and mitochondrial activities and at higher concentrations (≥75 µg/mL) increased ROS levels and modulated inflammation by reducing pro-inflammatory cytokines (IL-6, TNF-α) while promoting pro-regenerative cytokines (IL-33 and TGF-β). Treatment with 50 µg/mL of EVs optimally stimulated wound closure and growth factor secretion (VEGF, BDNF, KGF, and IGF) and upregulated ECM-related gene expression (type I and III collagen and fibronectin) [84].

### 7.5. Role of PMSC-Derived EVs in Macrophage M2 Polarization and in Reducing Inflammation in Diabetic Wound Healing

Diabetic wounds are usually in a prolonged pro-inflammatory M1 phase, preventing the progression to the pro-regenerative M2 phase necessary for tissue repair [85]. The immune-privileged properties that PMSC-EVs possess are unmatched immunomodulatory capability [86,87] and potent anti-inflammatory mediators, excelling at shifting the polarization of macrophages from the destructive M1 phenotype to the anti-inflammatory, pro-regenerative M2 phenotype [80,85]. This phenotypic shift is associated with a reduction in local inflammation and enables the initiation of tissue remodeling and angiogenesis [88].

### 7.6. Role of PMSC-Derived EVs in Keratinocyte Migration in Diabetic Wound Healing

PMSC-EVs exhibit superior pro-migratory effects due to their higher content of angiogenic and epithelial regulatory microRNAs, stronger anti-inflammatory activity, and enhanced resilience under diabetic conditions. Keratinocyte migration is essential for re-epithelialization, which is a key step in wound closure in diabetic wounds. This process is impaired due to hyperglycemia-induced oxidative stress, reduced growth factor signaling, chronic inflammation, and cytoskeletal dysfunction, resulting in delayed healing and chronic ulcers. PMSC-EVs is a potent regulator of keratinocyte migration through their rich cargo of miRNAs, growth factors, and signaling molecules [84,89]. PMSC-EVs enhance keratinocyte migration by activating the PI3K–AKT signaling pathway through delivery of miR--150-5p-which suppresses PTEN and promotes cell survival and motility. This pathway facilitates cytoskeletal reorganization and lamellipodia formation, enabling directional migration [90]. In addition, PMSC-EVs deliver microRNAs such as miR-31 and miR-23a/27b, which regulate actin dynamics and enhance cellular motility [91]. PMSC-EVs amplify EGFR and MAPK/ERK signaling, promoting keratinocyte proliferation and migration through delivery of EGF-like ligands and activation of downstream pathways. Activation of the Wnt/β-catenin pathway further enhances the transcription of genes involved in epithelial regeneration and migration [92]. PMSC-EVs also regulate cell adhesion and extracellular matrix remodeling by increasing integrin expression and activating focal adhesion kinase (FAK) while upregulating matrix metalloproteinases (MMP-2 and MMP-9), facilitating keratinocyte movement through the wound matrix [43] and reducing oxidative stress and inflammation via the activation of Nrf2 signaling and suppression of pro-inflammatory cytokines, creating a microenvironment conducive to migration. Beyond direct effects on keratinocytes, PMSC-EVs indirectly promote migration by modulating other wound healing cells. They induce macrophage polarization toward the pro-healing M2 phenotype and enhance fibroblast activity, both of which support epithelial migration and tissue regeneration.

### 7.7. Role of PMSC-Derived EVs in Reducing Oxidative Stress and Enhancing Neuroprotection in Diabetic Wound Healing

Another important value of EVs derived from MSCs and PMSCs is the ability to relieve oxidative stress [43,93]. Moreover, PMSCs harbor a unique cargo of antioxidant enzymes and specific miRNAs with neuroprotective and antioxidant capacities that neutralize ROS [86,87,88,90]. Studies have found that the antioxidant enzyme superoxide dismutase (SOD) can get encapsulated in PMSC-derived EVs and results in reduced oxidative stress and neutrophil inflammatory response [94]. Additionally, PMSC-derived EVs have been shown to carry various miRNAs that reduce oxidative stress such as miRNA-21, miRNA-34a, and miRNA-126 [95,96,97]. Also, miRNA-21 has been shown to directly target SOD, or NF-κB, whereas miRNA-34a is a direct regulator of NADPH oxidase (NOX), SIRT1, or p53 [98]. These specific antioxidant effects help to regulate excessive oxidative stress, further promote angiogenesis and re-epithelialization, and enhance wound healing. Small EVs (exosomes) derived from PMSCs modulate miR-126 to ameliorate hyperglycemia-induced inflammation [99]. Further research is needed to further our understanding of the gene sequencing changes of miRNAs in wound tissues after the application of PMSC-EVs. In preclinical models of multiple sclerosis, PMSC-EVs have demonstrated superior efficacy by reducing DNA damage and directly promoting remyelination [98,100]. They induce endogenous oligodendrocyte precursor cell (OPC) differentiation into mature myelinating cells, a capability that distinguishes PMSC-EVs as a novel therapeutic candidate for neurodegenerative diseases [86]. UC-MSC EVs promote the growth and migration of dermal fibroblast cells. In in vitro culture, dermal fibroblasts could be promoted to nerve cells and secrete nerve growth factors when stimulated by small EVs (exosomes). During the repair process, UC-MSC-EVs accelerated the recruitment of fibroblasts at the site of trauma and significantly enhanced cutaneous nerve regeneration in vivo. Interestingly, it was found that UC-MSC-EVs could promote wound healing and skin regeneration by recruiting fibroblasts, stimulating them to secrete nerve growth factors (NGFs) and promoting skin nerve regeneration [101].

### 7.8. Role of Proteins in PMSC-Derived EVs in Diabetic Wound Healing

In a study to quantify proteins in PMSCs and their exosomes through a label-free proteomics method, functional enrichment analysis was carried out for the differentially expressed proteins (DEPs), aiming to illustrate the potential function of the proteins in PMSC-derived exosomes for wound healing. Their results showed that the proteins enriched in PMSC exosomes were significantly involved in extracellular matrix organization, epithelium morphogenesis, cell growth, adhesion, proliferation, and angiogenesis. The protein–protein interaction analysis (PPI) was characterized by hub proteins such as POSTN, FN1, SPARC, TIMP1, SERPINE1, LRP1, and multiple collagens [37]. They conclude that expression of exosomal proteins derived from PMSCs reveals diverse functions of regeneration and tissue remodeling based on proteomics analysis and was strongly associated with wound healing [37].

Phospholipids contribute to EV membranes and function to hold barriers between the extra- and intracellular environments [98]. PMSCs help promote this function. It is important to note that PMSC-EVs contain specific cargos with proteins, miRNA, and lipids while tackling hemostasis, inflammation, proliferation, and remodeling, making it a perfect option for wound healing [102].

### 7.9. Molecular Mechanisms and Signaling Pathways Involved in PMSC-Derived EVs’ Improvement of Diabetic Wound Healing

PMSC-derived-EVs also aid in many pathways that promote wound healing. The Phosphoinositide 3-kinase/Protein Kinase B (PI3K/Akt) signaling pathway is a central regulator of cellular survival, proliferation, and angiogenesis. In a diabetic microenvironment, high glucose and oxidative stress typically inhibit this pathway, leading to endothelial cell apoptosis and vascular injury. PMSC-EVs act as potent biological triggers that reactivate this pathway through the delivery of specific molecular cargo and lead to reduced DNA damage in oligodendroglia populations and increased myelin regeneration. In vitro data demonstrate that PMSC-EVs promote myelin regeneration by inducing endogenous oligodendrocyte precursor cells to differentiate into mature myelinating oligodendrocytes [98]. In addition, PMSCs enhance the PI3K/Akt Pathway, which helps promote cell survival proliferation and angiogenesis and is deregulated in diabetic patients. miRNA-126 and VEGF are key factors that are inside the EVs’ cargo and promote this pathway [103].

EVs also help to modulate the Wnt/β-catenin pathway, thereby increasing fibroblast production and deposition, promoting dermal production [104,105]. Activation of this pathway has been shown to increase dermal production in mouse models [105]. One of the advantages of using EVs in wound healing is the ability to reduce inflammatory properties. The EVs reduce pro-inflammatory cytokines and suppress the NF-Kb pathway, causing a reduction in TNF-α, IL-1β, and IL-6 and reducing inflammation at wound sites [106].

## 8. Experimental Evidence and Preclinical Studies of MSC-Derived EVs in Diabetic Wound Healing

There have been numerous in vitro and in vivo studies using EVs to enhance wound healing in conditions that mimic diabetes. Placental MSC-derived EVs have been used as a method of enhanced wound healing in diabetic keratinocytes and animal models, as summarized below.

### 8.1. Use of PMSC-Derived EVs for In Vitro Studies to Enhance Wound Healing

One study focused on using immortalized keratinocyte cell lines, like HaCat cells, to examine wound healing by creating scratches using sterile tips and cells continually cultured without fetal bovine serum [107]. PMSCs and their derived EVs were safely added to HaCat cells to examine if there was an increased effect on wound healing. The effects were examined for 48 hours; the wound scratch was significantly smaller in the cell lines treated by PMSC-derived EVs versus controls [107]. This experiment demonstrated that PMSC-derived-EVs significantly enhanced the proliferation and migration of epidermal cells, facilitating wound closure. Another study showed that EV-treated keratinocytes exhibited increased migratory capacity in scratch assays and attributed to the upregulation of integrins and matrix metalloproteinases (MMPs) [102]. There are other studies conducted that yielded similar results, showing the cell lines treated with small EVs (exosomes), whether they are of placental, umbilical cord, or adipose tissue, had a significant impact on wound healing [108]. These cell lines showed a significant improvement in wound closures, confirming that EVs may be effective as therapeutic agents in enhancing wound healing.

### 8.2. Use of Placental MSC-Derived EVs In Vivo to Enhance Wound Healing

In vivo, animal studies were conducted using diabetic rat/mouse models. Thick cutaneous wounds were created in the rats. These rats were split into control and placental MSC treatment groups. Over 16 days, the experimental groups had a drastic increase in wound closure and healing, while the control did not show as much of an effect. It is important to note that the placental MSC group had a faster wound healing time compared with the control [109].

Another study was conducted on non-diabetic rat models. These groups were treated with either PMSC-derived EVs, adipose-derived MSCs (AMSCs), or a control [110]. Each experimental group had a drastic increase in wound healing time compared to the control. However, the placenta-derived MSC and EV groups had a greater healing process compared with the adipose-derived MSCs, indicating that a placenta source could be a more viable option as a diabetic wound healing agent. The improvement included the rate of healing, the quality of healing, higher collagen production, endothelial cell growth, and well-formed appendages. However, more studies are needed to further support these results.

In conclusion, several preclinical in vitro and in vivo studies supported the potential use of PMSC-derived EVs to enhance wound healing, especially under diabetic conditions. These studies have proven the superiority of these PMSC-EVs over EVs from other sources, paving the way for clinical studies to explore the safety and efficacy of those EVs as solutions for diabetic wound healing problems.

### 8.3. Use of Hydrogels Loaded with PMSC-EVs for Diabetic Wound Healing

MSC-EVs have a short half-life, and there is a need for repeated applications for the treatment to be efficient for regenerative purposes and to be effective for the healing process. Therefore, in recent years, different types of biomaterials such as hydrogels have been developed and tested for their progressive release and creation of a suitable microenvironment that favors healing [87]. Synthetic and natural polymers have been used to make some of these hydrogel dressings. The following are examples of hydrogels used clinically: hydrogels of hydrophilic polymers, hydrocolloid hydrogels mixed with adhesive materials and synthetic rubber, alginate polysaccharide hydrogels, and silicone or polyurethane hydrogel foams [87,111]. The development of multifunctional hydrogels is a promising strategy and has unique properties of bactericidal and wound healing effects that can accelerate diabetic wound healing and may be an important part of future diabetic wound management [18]. AD-MSC- and UC-MSC-derived EVs have been incorporated into hydrogels, and these EVs can be preserved up to 12 weeks without significant loss of functionality [112,113,114]. These hydrogels loaded with EVs increased the production of collagen III during healing, favoring the formation of scar-free wounds [113,114,115]. These hydrogels also decreased the inflammatory response by downregulating TNF-α and IL-6, as well as promoting polarization from M1 into M2 macrophages [113,114]. Hydrogel loaded with UC-MSC-EVs were evaluated in a streptozotocin-induced diabetic rat model of scarring [114]. These EV-loaded hydrogels protected the biological activity of UC-MSC-EVs, increasing angiogenesis and granulation tissue formation, accelerated wound healing, and upregulated the expression of VEGF and TGFβ-1 relative to treatment with MSC-EVs or hydrogels alone [114]. Thus, this hydrogel biomaterial-based EV therapy may represent a new therapeutic approach for cutaneous regeneration of chronic diabetic wounds.

## 9. Challenges and Limitations of Potential Clinical Use of Placental Mesenchymal Stem Cell-Derived Extracellular Vesicles (PMSC-EVs) in Diabetic Wound Healing

Studies have shown that the use of MSC-derived EVs from many sources such as placental, umbilical cord, adipose tissue, etc., has been linked to enhanced and effective wound healing in in vitro and in vivo diabetic models. PMSC-EVs offer a promising and acellular alternative to BM- and AD-MSC-EVs for diabetic wound healing. Their unique perinatal origin and non-invasive and ethically acceptable acquisition provide them with distinct clinical and biological edges. PMSC-EVs’ immunomodulatory and angiogenic signaling required for placental development and fetomaternal tolerance effectively neutralize the persistent inflammation, high oxidative stress, and dysfunctional angiogenesis that characterize diabetic wounds. With their ability to restore vital repair processes and overcome the hyperglycemic stalemate, PMSC-EVs are positioned to catalyze an important shift in the management of diabetic wound healing. EVs are rich in proteins, miRNAs, and lipids that all support the physiological processes that are downregulated in diabetic patients. Preclinical studies showed a promising alternative to enhance wound healing and support the development of clinical studies on humans. There have been some studies on diabetic foot ulcers showing increased wound healing [76,116]. These may serve as more effective cell-free therapeutic options compared with current treatments for diabetic wounds.

One of the major challenges in clinical application of EVs is the standardization of large-scale EV production. The current heterogeneity of EV extraction and preparation and therapeutic properties pose an issue that needs to be resolved prior to production for clinical applications. EV subpopulations can have various amounts of proteins and cargo properties that may affect their clinical effect. In addition, methods of delivery pose questions as to what the best possible solution to protect the EVs from damage when delivering the EVs to the wound site. Protection of the EVs and their respective cargo is necessary to make them as effective as possible in wound healing [117]. Additionally, compliance with the FDA’s current Good Manufacturing Practices (cGMP) is necessary to have EVs used for therapeutic applications. This ensures that there is no contamination or defect and makes sure it is safe for clinical applications in the skin of humans.

Ongoing research should focus on optimizing standardized isolation methods and developing smart delivery system such as smart hydrogels to optimize and control the release of this potent cell-free therapy [87]. One possibility for enhancing the efficiency of EVs could be engineering EVs to be loaded with specific proteins, RNAs, and miRNAs, making them more effective in wound healing. A study using lncRNA-H19 on artificial EV-mimetic nanovesicles has shown accelerated wound healing in rat models. This is one example of how modifying EVs can be useful for wound healing [118]. MSC-derived EVs have large and small EVs that may affect their potency and may have differences in their clinical effectiveness. Developing standards to make EVs uniform as therapeutic agents will aid in using them clinically. Human Telomere Reverse Transcriptase (hTERT) is an enzyme that lengthens telomeres and further reduces cellular senescence in cells [119,120]. There is potential of depositing the hTERT enzyme in PMSC-derived EVs, which would increase proliferative activity, reduce senescence activity, and stabilize the phenotype, making the derived EVs more homogenous and better for therapeutic applications.

Ziegler et al. indicated that unmodified EVs may exhibit poor targeting efficacy, leading to the necessity of engineered EVs for their greater promise in clinical applications by mediating good targeting efficacy, exhibiting a targeting peptide that allows them to specifically target a specific organ or even cell type, thus avoiding accumulation in undesired locations and increasing the potency of the treatment [120,121]. The engineered EVs can be artificially pre-loaded with any necessary biomolecular cargo or even therapeutic drugs to treat a variety of human diseases [120,121]. Ma et al. recently summarized the steps needed to replace or modify dysfunctional cells with healthy cells or engineered derivatives to offer disease reversal and cure [122]. The bioengineering strategies help with mass production of EVs, EV modification, EV membrane perturbation, and cargo encapsulation. The bioengineering of these cells offers therapeutic benefits similar to cell transplants without the biosafety risks; may overcome the limited production, inadequate therapeutic loading, and poor targeting efficiency; and enhance their effectiveness [122]. The translational application of engineered EVs is quite promising, but there are multiple challenges that have to be addressed such as finding the most effective methods of mass production of EVs necessary for large-scale clinical applications and developing quicker safe methods for EV isolation with good yields [121]. It is also important to find the most efficient methods of long-term storing of EVs, feasible and cost-effective large-scale transportation from a laboratory to hospitals or clinical settings for use, and easy EV recovery after storage to maintain their biodistribution [123]. However, more research must be performed to determine the ways EVs can be isolated quickly, cost effectively, and in a high-yield fashion before EVs can have widespread clinical applications.

## 10. Conclusions

In conclusion, diabetes wounds are a major challenge due to delayed healing that ranges from deregulated angiogenesis with a lack of endothelial cells reaching the wound site to downregulated VEGF, increased inflammation, oxidative stress, and fibroblast dysfunction. Most treatments failed to address these issues, but PMSC-derived EVs are emerging as a possible alternative. PMSC-derived EVs’ cargo contains an abundance of miRNA, proteins, and lipids that may combat these downregulated functions. These miRNA and proteins in the EVs’ cargos help to promote angiogenesis by delivering VEGF and HIF-α and provide an influx of blood supply to the wounds. These proteins also help to reduce inflammation by inhibiting the NF-Kb pathway, fibroblast regulation, increasing collagen production, and helping with dermal regulation in diabetic keratinocytes. MSC-derived EVs show a promising alternative therapy for diabetic wound healing. However, several challenges still hinder the clinical translation of EV-based therapies, including the need for scalable production methods, more efficient delivery systems, and overcoming regulatory obstacles [124]. To advance these therapies, future research should prioritize optimizing EV isolation processes, engineering EVs to boost their therapeutic efficacy, and incorporating them with biomaterials to develop more robust wound healing platforms [124]. Tackling these issues will be essential for establishing PMSC-derived EVs as a safe, effective, and practical treatment option for diabetic wounds. More research is needed to examine their therapeutic potential in humans. MSC-derived EVs are complex, and challenges such as standardization are halting the process of making this a readily available treatment for diabetic patients. PMSC-derived EVs target cellular and molecular mechanisms that are hindered by diabetes to promote effective and efficient wound healing.

## Data Availability

Data sharing is not applicable.
